# Characterization of resistance mechanisms of *Enterobacter cloacae* Complex co-resistant to carbapenem and colistin

**DOI:** 10.1186/s12866-021-02250-x

**Published:** 2021-07-08

**Authors:** Shixing Liu, Renchi Fang, Ying Zhang, Lijiang Chen, Na Huang, Kaihang Yu, Cui Zhou, Jianming Cao, Tieli Zhou

**Affiliations:** 1 Department of Clinical Laboratory, The First Affiliated Hospital of Wenzhou Medical University, Wenzhou, 325035 China; 2 Department of Laboratory Medicine, The First Affiliated Hospital, College of Medicine, Zhejiang University, Hangzhou, 310003 China; 3 School of Laboratory Medicine and Life Sciences, Wenzhou Medical University, Wenzhou, 325035 China

**Keywords:** *Enterobacter cloacae* Complex, Carbapenem, Colistin, Co-resistance

## Abstract

**Background:**

The emergence of carbapenem-resistant and colistin-resistant ECC pose a huge challenge to infection control. The purpose of this study was to clarify the mechanism of the carbapenems and colistin co-resistance in *Enterobacter cloacae* Complex (ECC) strains.

**Results:**

This study showed that the mechanisms of carbapenem resistance in this study are: 1. Generating carbapenemase (7 of 19); 2. The production of AmpC or ESBLs combined with decreased expression of out membrane protein (12 of 19). *hsp60* sequence analysis suggested 10 of 19 the strains belong to colistin hetero-resistant clusters and the mechanism of colistin resistance is increasing expression of *acrA* in the efflux pump AcrAB-TolC alone (18 of 19) or accompanied by a decrease of affinity between colistin and outer membrane caused by the modification of lipid A (14 of 19). Moreover, an ECC strain co-harboring plasmid-mediated *mcr-4.3* and *blaNDM-1* has been found.

**Conclusions:**

This study suggested that there is no overlap between the resistance mechanism of co-resistant ECC strains to carbapenem and colistin. However, the emergence of strain co-harboring plasmid-mediated resistance genes indicated that ECC is a potential carrier for the horizontal spread of carbapenems and colistin resistance.

**Supplementary Information:**

The online version contains supplementary material available at 10.1186/s12866-021-02250-x.

## Background

*Enterobacter cloacae* complex (ECC) belongs to the *Enterobacter* genus, which exists widely in nature and is also part of the common bacterial flora of the human gastrointestinal tract. In the past few decades, ECC was one of the most common pathogens in hospitals, often causing various infections, such as pneumonia, urinary tract infections, and sepsis [1]. Due to the widespread use of antibiotics, multi-drug resistant (MDR) ECC strains have emerged and spread around the world and ECC infection accounts for 65–75% of *Enterobacter* infections, which was called “ESKAPE” pathogen with five other common pathogens (*Enterococcus faecalis, Staphylococcus aureus*, *Klebsiella pneumoniae*, *Acinetobacter baumannii*, *Pseudomonas aeruginosa*) [2, 3].

*hsp60*, known as the *groEL* homologue coding for the 60-kDa heat shock protein, has been successfully applied for the classification of many bacteria and turned out to be useful for the phylogenetic analysis of Enterobacter in the present study [4–10]. The ECC is divided into 13 clusters (C-I to C-XIII) by analysis for partial sequencing of the *hsp60* gene, three of which (C-III, VI and VIII) being the most frequently recovered from human clinical samples [11].

ECC is intrinsically resistant to penicillins and first- and second-generation cephalosporins due to low-level expression of chromosomal *ampC* genes encoding an inducible AmpC-type cephalosporinase [12]. When ECC is exposed to β-lactam drugs for a long period of time, it can further lead to the highly induced phenotypes of AmpC cephalosporin, thus producing resistance to third-generation cephalosporins [13]. Besides, the acquisition of a variety of plasmids mediated extended-spectrum β-lactamase (ESBL) genes conferred ECC resistance to most β-lactam drugs, making the treatment more difficult. Literature has shown that the resistance of *Enterobacter cloacae* to carbapenem can be mediated by AmpC expression and membrane permeability (the decrease in or loss of the outer membrane proteins OmpF and OmpC) changes, but it may be more common to obtain carbapenemase gene transferred by plasmids [14, 15]. The plasmid-mediated mechanism significantly increases the spread of carbapenem resistance, while further limiting the choice of effective antibacterial drugs.

However, several recent studies have found that ECC strains carry carbapenemase genes while being resistant to colistin [16–18]. And ECC co-resistant to carbapenem and colistin had been reported scattered in India, the United States, China, and Japan and an outbreak of 18 co-resistant ECC strains in France from 2015 to 2017 [16, 18–22]. Colistin is an old antibiotic that has been refocused in recent years. Because of the good activity against Gram-negative bacteria, it is considered to be one of the last-line antimicrobials for treatment of MDR Gram-negative bacteria [23]. According to previous reports, ECC may acquire colistin resistance by plasmid-mediated gene *mcr*, small protein gene *ecr*,or two-component systems *phoPQ*, *pmrAB*, which lead to modification of lipid A [17, 24–27]. The emergence of carbapenem-resistant and colistin-resistant ECC will undoubtedly pose a huge challenge to infection control. And the mechanism of carbapenem and colistin resistance mediated by movable elements also increases the risk of widespread transmission.

The purpose of this study is to clarify the mechanism of the carbapenems and colistin co-resistance in ECC strains. We collected co-resistant ECC clinical isolates from a regional medical center, and performed this study through methods such as phenotype testing, gene identification, relative expression detection, and mass spectrometry analysis, to provide further understanding for the resistance development of ECC strains.

## Results

### Results of antimicrobial susceptibility testing and efflux inhibitors assay

**Fig. 1:**
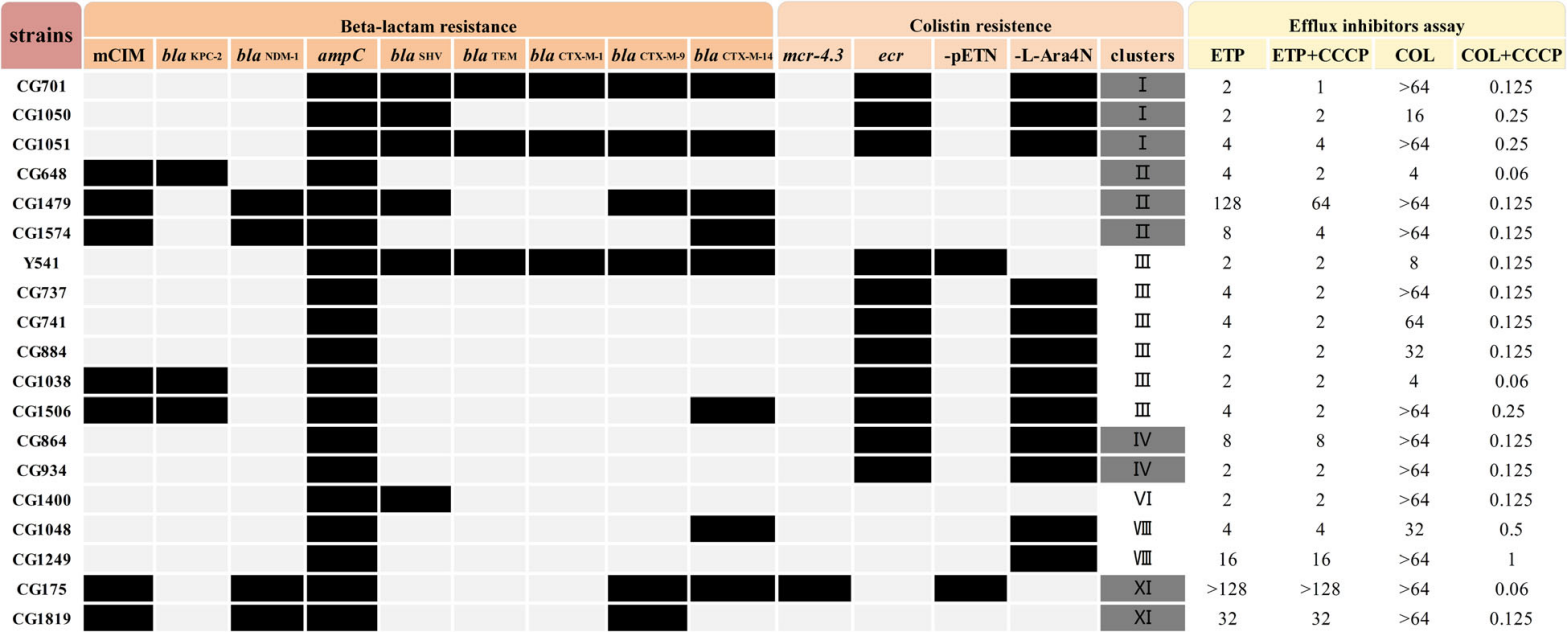
Results of resistance-related mechanisms of 19 carbapenems and colistin co-resistant ECC strains. The black squares in the figure represent positive results, the light gray squares represent negative results, and the dark gray squares represent cluster-dependent colistin resistance. And the unit of MIC values in the efflux inhibitors assay is μg/mL. mCIM, modified carbapenem inactivation methods; −pETN, phosphoethanolamine; −L-Ara4N, 4-amino-4-deoxy-L-arabinose; CCCP, carbonyl cyanide m-chlorophenylhydrazone; ETP, ertapenem; COL, colistin

Antimicrobial susceptibility testing was used to determine the minimum inhibitory concentrations (MICs) of Carbapenem and Colistin. As shown in Table S2, all 19 ECC strains were resistant to ertapenem (MIC_50_ = 4 μg/mL) and colistin (MIC_50_= > 64 μg/mL). Besides, 5 ECC strains were resistant to meropenem, 6 ECC strains were resistant to imipenem, and 4 of them were resistant to 3 carbapenems. The results obtained from the efflux inhibitors assay were shown in Fig. 1 and Table S3. In the presence of efflux pump inhibitors carbonyl cyanide m-chlorophenylhydrazone (CCCP) or omeprazole, the MICs of colistin to 19 ECC strains or 17 ECC strains decreased significantly and returned to susceptible respectively, while almost all inhibitors of efflux pump activity showed no effect on the MICs of ertapenem. Taken together, these results suggest that there is an association between colistin resistance of ECC strains and efflux pump.

### Prevalence of carbapenemase, ESBLs and AmpC cephalosporinase

As summarized in Fig. 1, 7 ECC strains were positive in modified carbapenem inactivation methods (mCIM), indicating that they are carbapenemase-producing strains (Fig. 1). Further identified the types of carbapenemase genes carried by these 7 ECC strains, including 3/19 (15.8%) *bla*_KPC-2_ gene and 4/19 (21.1%) *bla*_NDM-1_ gene. In addition to carbapenemase encoding genes, ESBL genes were detected in 11 of 19 ECC strains, with *bla*_CTX-M-14_ the most prevalent (*n* = 8, 42.1%), followed by *bla*_CTX-M-9_ (*n* = 6, 31,6%), *bla*_SHV_ (*n* = 6, 31.6%), *bla*_TEM_ (*n* = 3, 15.8%), *bla*_CTX-M-1_ (*n* = 3, 15.8%) (Fig. 1). AmpC cephalosporinase gene *ampC* were detected in all 19 ECC strains (Fig. 1).

### Genetic clusters based on *hsp60* sequence analysis

To investigate the species distribution among 19 of the strains, we performed sequence analysis of the *hsp60* gene. *hsp60* sequence analysis suggested they were divided into 7 clusters and genetic cluster belonging to the cluster III were predominant, accounting for 31.6% (6/19), following 3, 3, 2, 2, 2, 1 of the clusters I, IV, VII and XI, respectively (Fig. 1).

### Relative expression of genes encoding outer membrane protein and efflux pump protein

**Fig. 2:**
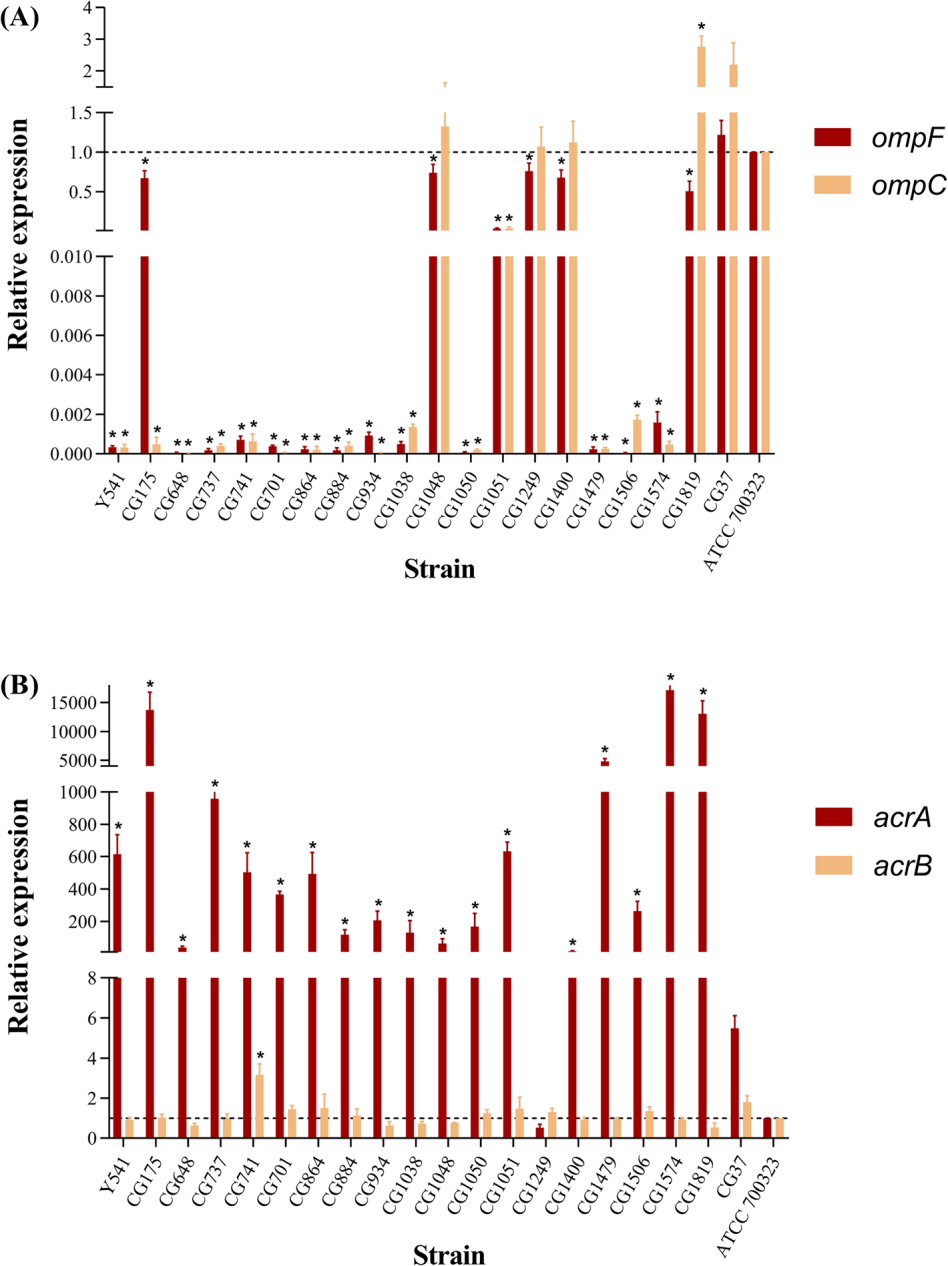
Relative expression of outer membrane protein gene *ompC*/*OmpF* and efflux pump protein gene *acrA*/*acrB*. **A** Expression of *ompC* and *OmpF*; **B** expression of *acrA* and *acrB*. CG37 is a clinical strain susceptible to carbapenem and colistin and ATCC700323 is the reference strain. *, *P* < 0.05 were considered to indicate statistical significance

To assess the relationship between carbapenem resistance and the expression of outer membrane proteins OmpC and OmpF and the relationship between colistin resistance and expression of efflux pump proteins AcrA and AcrB, 19 ECC strains co-resistant to carbapenem and colistin, 1 carbapenem and colistin co-susceptible ECC strain CG37 and *Enterobacter cloacae* ATCC 700323 were used. As shown in Fig. 2, compared with ATCC 700323, the expression level of *ompC* was reduced in 15 co-resistant ECC strains (*P* < 0.05). Furthermore, the expression of *ompF* was deficient in all 19 co-resistant ECC strains (*P* < 0.05). The efflux pump gene *acrA* was found to be significantly overexpressed in 18 co-resistant ECC strains (*P* < 0.05). Conversely, the *acrB* gene was not found to be significantly overexpressed in co-resistant ECC strains (except CG741).

### Prevalence of *mcr*, *ecr* genes and structural modification of wild-type lipid a

**Fig. 3:**
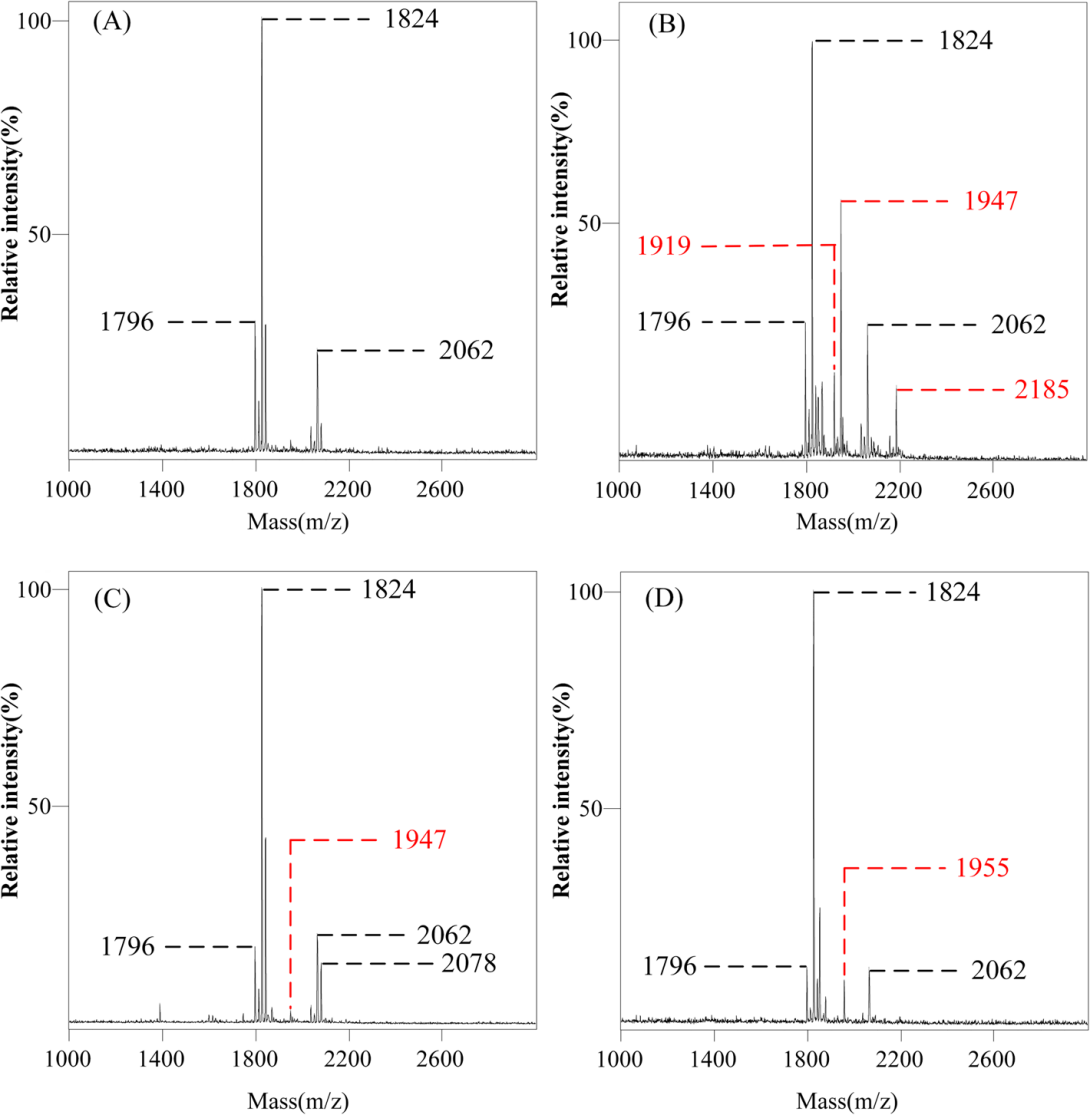
Results of MALDI-TOF mass spectrometry of lipid A. There are 4 different types of MS profile as shown by (**A**, **B**, **C**, **D**). The black labeled ion peak is the original peak or modified peak not related to colistin resistance, and the red labeled ion peak is the modified peak related to colistin resistance

**Fig. 4:**
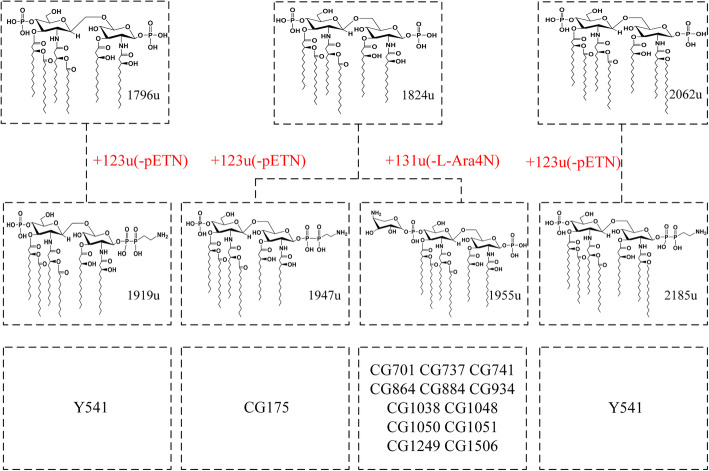
Lipid A structures with corresponding m/z values found in ECC isolates

To investigate whether the colistin resistance of the ECC strains in this study is related to the modification of lipid A, we detected the relevant resistance genes and analyzed the ion peak spectrum of lipid A using matrix-assisted laser desorption/ionization time of flight mass spectrometry (MALDI-TOF MS). From Fig. 1 we can see that the ECC strain CG175 harboring *mcr-4.3*, which belongs to the plasmid-mediated *mcr* family gene. Moreover, a novel transmembrane protein gene *ecr* was found in other 11 ECC strains (Y541, CG737, CG741, CG701, CG864, CG884, CG934, CG1038, CG1050, CG1051, CG1506). The structures of lipid A in ECC strains in this study showed diversity (Fig. 3 and Fig. 4). Among them, colistin-susceptible ATCC 700323 possessed wild-type lipid A with three ion peaks of *m/z* 1796, 1824, and 2062, respectively (Fig. 3a). Wild-type lipid A was also present in 19 co-resistant ECC strains, of which the ion peak of *m/z* 1824 is predominant. Interestingly, 14 of the 19 ECC strains contained modified lipid A related to colistin resistance. Taken together, the ion peak spectrum of 14 co-resistant ECC strains can be divided into 3 different modes (Fig. 3b, Fig. 3c. Figure 3d), including 4 ion peaks of *m/z* 1919, 1947, 1955, and 2185. From the data in Fig. 4, the groups that modify wild-type lipid A and cause resistance to colistin are phosphoethanolamine (pETN, *m/z* 123) and 4-amino-4-deoxy-L-arabinose (−L-Ara4N, *m/z* 131).

## Discussion

ECC can adapt to the environment quickly and obtain resistance to various antibacterial drugs by inducing resistance determinants and gaining exogenous resistance genes. The emergence of MDR ECC poses a huge challenge for effective clinical treatment.

Carbapenem is an atypical β-lactam antibiotic with the broadest resistance spectrum and has the best antibacterial effects at present. It also maintains good susceptibility to *Enterobacteriaceae* carrying ESBLs or overexpressing AmpC-type cephalosporinase, and is the choice for clinical control of MDR ECC infections [28, 29]. However, with the widespread use of carbapenem, the number of carbapenem-resistant ECC clinical strains is increasing, which can lead to failure of clinical anti-infection treatment. As mentioned in the literature review, the main mechanisms that mediate the resistance of ECC strains to carbapenem are: 1. Generating carbapenemase; 2. The production of AmpC or ESBLs combined with a loss or decreased expression of out membrane protein [30, 31]. In addition, studies have found that the susceptibility of *Escherichia coli* and *Enterobacter cloacae* to carbapenem can be increased when the AcrAB-TolC efflux pump is inhibited [32, 33].

Colistin is an “old” antibiotic, which has been re-applied to the clinic because of its good antibacterial activity against MDR gram-negative bacteria. In fact, it is the last line of drug for the treatment of serious infections caused by MDR gram-negative bacteria. As reported by Mirelis, the total resistance rate of *Enterobacteriaceae* to colistin was 0.67%, but the resistance rate of *Enterobacter cloacae* (4.2%)was much higher than that of *Escherichia coli* and *Klebsiella pneumoniae* [34]. The resistance mechanism of ECC strains to colistin can be mediated by the *mcr-1* gene carried by the plasmid [24, 35, 36], and may also be related to the mutations of the two-component system encoded by *pmrAB* and *phoPQ* [17]. Recently, Zong et al. found that a novel small transmembrane protein Ecr may mediate the heterogeneous resistance of ECC to colistin through the PhoPQ two-component system [25], but the distribution of *ecr* gene in ECC strains and its correlation with colistin resistance still need further study. In addition, François et al. considered that the colistin hetero-resistance appeared cluster-dependent in the ECC: strains from clusters I, II, IV, VII, IX, X, XI, and XII were usually hetero-resistant and for some cluster V and VIII strains, a small proportion (< 10^− 7^) of cells appeared resistant when tested by population analysis profiling [10]. In reviewing the literature, colistin resistance mediated by *mc*r series genes or mutations in *pmrAB* and *phoPQ* is related to structural modification of lipid A of ECC strains, such as phosphoethanolamine (pETN) or 4-amino-4-deoxy-L-arabinose (−L-Ara4N). The modification of wild-type lipid A can reduce the potential of the outer membrane in *Escherichia coli* and reduce the affinity of colistin to the outer membrane [37, 38]. Furthermore, the overexpression of efflux pump AcrAB-TolC may also lead to the resistance of ECC strains to colistin [39]. And the clusters I, II, IV, VII, IX, X, XI and XII were hetero-resistant to colistin in ECC [10]. In recent years, carbapenems and colistin co-resistant ECC strains have been found in multiple regions around the world [16–19, 21, 40], which will further increase the difficulty of treating MDR ECC infection. And some of the co-resistant ECC strains have the ability to spread drug resistance gene horizontally, which posing a huge threat to public health.

The current study found that 7 out of 19 carbapenems and colistin co-resistant ECC strains carried the carbapenemase gene, of which 3 strains carried *bla*_KPC-2_ and 4 strains carried *bla*_NDM-1_. It can be found that the ECC strains carrying the carbapenemase gene are all resistant or intermediary to meropenem, imipenem, and ertapenem, but most of the ECC strains that do not carry the carbapenemase gene were only resistant to ertapenem in a low level, and were susceptible to meropenem and imipenem. This finding is in agreement with CLSI guidelines which suggested that resistance of *E. cloacae* to imipenem and meropenem is usually related to the carbapenemase gene [41]. This study found that the predominant mechanism of carbapenem resistance in ECC strains is not generating carbapenemase. The results of this study showed that AmpC or ESBLs production combined with decreased expression of out membrane protein confer low-level resistance to ertapenem in ECC strains that do not produce carbapenemase. In addition, no correlation was found between the efflux pump AcrAB-TolC and carbapenem resistance of ECC strains in this study.

This study found that 11 (57.9%) of the 19 ECC strains carry the *ecr* gene, which is thought to be related to colistin heterogeneity resistance by regulating lipid A modification. Further analysis of lipid A by MALDI-TOF mass spectrometry revealed that all ECC strains carrying the *ecr* gene undergo lipid A modification, suggesting that *ecr* may mediate colistin resistance in this study. Interestingly, of the 11 strains carrying the *ecr* gene, except for the wild-type lipid A of Y541, which was modified with phosphoethanolamine (pETN), and the remaining strains were all modified with 4-amino-4-deoxy-L-arabinose (−L-Ara4N). We speculate that Y541 may be involved in other colistin resistance mechanisms, which need further study. The results of the efflux inhibitor assay showed only CCCP and omeprazole had a significant effect on the MICs of colistin. CCCP is a strong uncoupling agent that acts as an efflux pump inhibitor against the SMR-type efflux pumps, MFS-type efflux pumps, MATE-type efflux pumps, and RND-type efflux pump. Omeprazole is a proton pump inhibitor. And reserpine as a pump inhibitor, itself is a substrate of certain efflux pumps, which can compete with antibacterial drugs to bind bacterial efflux pump proteins, and mainly inhibits MFS- and ABC-type efflux pump. So the results of the efflux inhibitor assay suggested that colistin resistance may be related to SMR-, RND-and MATE-type efflux pump. And the expression of the efflux pump genes *acrB* showed that except for the strain CG741, the expression levels of *acrB in* other ECC strains did not increase significantly (*P* > 0.05). However, the expression levels of *acrA* gene in 18 ECC strains increased in varying degrees (*P* < 0.05). This result is different from telk*e* et al. in 2017 report that the regulation of *sosRS* can simultaneously upregulate expression of *acrA* and *acrB* to induce heterogeneous resistance in *Enterobacter cloacae* [39]. This inconsistency may be due to *acrA, acrB* and *tolC* genes are not co-regulatory genes. Their expression is usually regulated by multiple levels of different regulatory factors [42–44]. The results of identifying clusters showed that 10 strains belonged to heterogeneous resistance clusters, including 3, 3, 2, 2 of the clusters I, II, IV, and XI, respectively, would partly explain the colistin resistance.

Notably, CG175 carries the *bla*_NDM-1_ gene along with the *mcr-4.3* gene. The genomic characteristics of this strain have been reported in the study of Chavda et al. [18]. This is the first *Enterobacter cloacae* isolate co-harboring *mcr-4.3* and *bla*_NDM-1_ reported in China. Although in the study by Chavda et al., it was found that *mcr-4.3* and *bla*_NDM-1_ were present in two separate plasmids (ColE plasmid and IncX3 plasmid) and *mcr-4.3* did not mediate colistin resistance. However, the emergence of this strain has sounded the alarm for clinical anti-infection treatment. Because ECC strains are widely distributed in the hospital environment, are potential carrier of mobile resistance genes.

## Conclusion

In summary, the main mechanisms of carbapenem resistance in this study are: 1. Generating carbapenemase; 2. The production of AmpC or ESBLs combined with decreased expression of out membrane protein. And the mechanism of colistin resistance is the increase of *acrA* in the efflux pump AcrAB-TolC alone or accompanied by a decrease of affinity between colistin and outer membrane caused by the modification of lipid A, as well as cluster-dependent colistin resistance.

## Methods

### Clinical isolates

Nineteen ECC isolates co-resistant to carbapenem and colistin were collected from the First Affiliated Hospital of Wenzhou Medical University in China, from 2011 to 2018. All isolates were identified by MALDI-TOF MS using a VITEK® mass spectrometer (BioMerieux, Lyons, France). The study was approved by the Biosafety Committee of the First Affiliated Hospital of Wenzhou Medical University and the data used in this study was anonymised before its use.

### Antimicrobial susceptibility testing

The MICs of carbapenems (meropenem, imipenem, and ertapenem) (Biokangtai Co., Ltd.) and colistin (Biokangtai Co., Ltd.) to the 19 ECC strains were performed by the broth microdilution in cation-adjusted Mueller-Hinton Broth (CAMHB). Briefly, bacteria were suspended in saline to 1/100 the turbidity of the 0.5 McFarland standard and the final bacteria concentration of each well was approximately at 7.5 × 10^5^ colony forming units (CFU)/mL. Serial two-fold dilutions ranging from 0.004 to 128 μg/ml for carbapenems, and 0.125 to 64 μg/ml for colistin were prepared in CAMHB 96-well microtiter plates. The results were quantified by measuring the OD_600_ value by the Multiskan FC Microplate Reader after incubation at 37 °C for 16–18 h. And the breakpoint of carbapenems and colistin for ECC was interpreted according to the guidelines of the Clinical and Laboratory Standards Institute (CLSI) [41] and European Committee on Antimicrobial Susceptibility Testing (EUCAST) [45] respectively. *Escherichia coli* ATCC 25922 was used as a control strain in this study.

### mCIM test

To screen for suspected carbapenemase production in the 19 ECC strains, the mCIM was performed. As recommended by CLSI, emulsify a 1 μL loopful of bacteria from an overnight blood agar plate in 2 mL trypsin soy broth (TSB). Add a 10-μg meropenem disk in each suspension using sterile forceps and incubate at 35 °C ± 2 °C in ambient air for 4 h ± 15 min. Remove the meropenem disk from each TSB-meropenem disk suspension and place it on the Mueller-Hinton agar (MHA) plate previously inoculated with the meropenem-susceptible *E.coli* ATCC 25922 indicator strain. Invert and incubate the MHA plates at 35 °C ± 2 °C in ambient air for 18–24 h. Carbapenemase positive when the antibacterial zone diameter of 6–15 mm or presence of pinpoint colonies within a 16–18 mm zone; Carbapenemase negative when the antibacterial zone diameter of ≥19 mm (clear zone).

### Polymerase chain reaction (PCR) detection for resistance genes

The genomic DNA of the 19 ECC strains was extracted using the Biospin Bacterial Genomic DNA Extraction kit (Bioflux, Tokyo, Japan). Using these DNA templates we detected the carbapenemase gene (*bla*_KPC_, *bla*_NDM_, *bla*_IMP_, *bla*_VIM_, *bla*_IMI_, *bla*_SPM_, *bla*_OXA-23_, *bla*_OXA-24_, *bla*_OXA-48_, *bla*_OXA-58_, *bla*_Nmc-A_, *bla*_FRI-1_, *bla*_BIC_, *bla*_GIM_, *bla*_SME_, *bla*_AIM_, *bla*_DIM_, *bla*_SIM_ and *bla*_GES_), extended-spectrum β-lactamase gene (*bla*_SHV_, *bla*_TEM_, *bla*_CTX-M-1_, *bla*_CTX-M-9_ and *bla*_CTX-M-14_), AmpC cephalosporinase gene *ampC*, colistin resistance-related genes (*mcr-1*, *mcr-2*, *mcr-3*, *mcr-4*, *mcr-5* and *ecr*). The primers used for PCR amplification were showed in Table S1. The positive PCR products were sequenced by Shanghai Genomics Institute Technology Co. Ltd. (Shanghai, China). And the sequences of the product were analyzed by BLAST searches against the NCBI database (www.ncbi.nlm.nih.gov/BLAST).

### Efflux inhibitors assay

In order to detect whether the efflux pump has an effect on drug resistance, MICs of ertapenem and colistin were determined in the presence or absence of efflux pump inhibitor: CCCP (8 μg/L, 1/4 MIC), reserpine (20 μg/L, 1/4 MIC), and omeprazole (200 μg/L, 1/4 MIC). CCCP and reserpine stock solutions use dimethyl sulfoxide (DMSO) as the solvent and omeprazole use 0.1% sodium hydroxide solution . When the efflux pump inhibitors were present, a reduction of at least four-fold in the MICs were considered indicative of efflux [46].

### Quantitative real-time PCR for outer membrane protein and efflux pump

Total RNAs of 19 ECC strains were extracted using the Bacterial RNA Miniprep Kit (Biomiga, Shanghai, China) according to the manufacturer’s instructions. Then, 500 ng of RNA was mixed with the reverse transcription system of the PrimeScript™ RT reagent Kit (Takara, Japan) to obtain 10 μL of cDNA. The expression levels of the outer membrane gene (*ompC*, *ompF*) and efflux pump gene (*acrA*, *acrB*) were performed by quantitative real-time PCR. The *rpoB* gene was selected as the internal gene. And the relative expression levels of four genes in 19 ECC strains were compared with the expression levels in co-susceptible *Enterobacter cloacae* ATCC 700323 and clinical strains CG37.

qPCR was performed using a CFX-96 touch™ Real-Time PCR system (Bio-Rad, California, USA) and TB Green Premix Ex Taq II (Tli RNaseH Plus) (2×) (Takara, Japan) with the specific primers (refer to Table S1). Then, 100 ng of cDNA was added to each sample as the template. Cycling conditions were as follows: 95 °C for 30 s followed by 40 cycles of 9 °C for 5 s and 60 °C for 20 s. A melting curve was performed after each run (raising 0.5 °C per second, from 65 °C to 95 °C). Each sample was run in triplicate and the means of Ct values were used for analysis. Quantification of the target genes was analyzed using the comparative threshold cycle 2^-ΔΔCt^ method. All experiments were repeated three times in triplicate independently.

### Lipid a characterization by MALDI-TOF MS

Lipid A was isolated by using an optimized large-scale protocol based on mild acid hydrolysis [47]. Overnight cultures (200 ml at 37 °C) in LB broth were harvested by centrifugation at 3220×*g* for 30 min. Bacterial pellets were washed with single-phase Bligh-Dyer mixture (chloroform/methanol/water, 1:2:0.8 [vol/vol]) and centrifuged at 3220×g for 15 min. The LPS pellets were suspended in sodium acetate buffer (50 mM [pH 4.5]) and incubated at 100 °C for 30 to 45 min. Reactions were moved into a two-phase Bligh-Dyer mixture (chloroform/methanol/water, 1:1:0.9 [vol/vol]) and centrifuged at 3220×g for 15 min. The lower phases were removed to clean tubes and dried using rotary evaporation. The dried samples contained whole-cell extracts of lipid A.

Dried lipid A samples were resuspended in 100 μl chloroform/methanol (1:1 [vol/vol]), and 3 μl 2,5-dihydroxybenzoic acid (DHB) matrix (20 mg/ml in TA30 solvent) was mixed with 3 μl lipid A. Aliquots of the mixture were spotted directly onto the well of the MALDI-TOF MS plate (ground steel). Mass spectra were recorded for optimal ion signals in negative-ion mode using a Bruker autoflex MALDI-TOF mass spectrometer (Bruker Daltonics Inc., Billerica, MA, USA). Data were acquired and processed by flexControl and flexAnalysis 3.4 (Bruker Daltonics Inc.).

### Identification based on *hsp60*

PCR analysis for partial sequencing of the *hsp60* gene was performed by a previously described protocol [11]. Briefly, primers h*sp60*-F (5′-GGTAGAAGAAGGCGTGGTTGC-3′) and *hsp60*-R (5′-ATGCATTCGGTGGTGATCATCAG-3′) were used for genomic amplification of a 341-bp fragment of the *hsp60* gene. A negative control containing all reagents except the target DNA (which was replaced by H2O) was included in each series. PCR was performed for 30 cycles by using the following conditions: 30 s at 94 °C for denaturation, 30 s at 57 °C for annealing, and 60 s at 72 °C for elongation. The positive PCR products were sequenced by Shanghai Genomics Institute Technology Co. Ltd. (Shanghai, China). And the sequences of the product were analyzed by BLAST searches against the NCBI database (www.ncbi.nlm.nih.gov/BLAST). A 272-bp fragment of the *hsp60* gene was obtained for the 19 strains, and the sequence of the fragment was compared to reference sequences from strains previously described in taxonomic studies [11] by using the Clustal W algorithm. Sequence comparisons were exported as an unrooted neighbor-joining tree with proportional branch lengths and strains were divided into 13 clusters.

### Statistical analysis

All data were analyzed using the GraphPad Prism v8.01 statistical software package (GraphPad Software, La Jolla, CA, USA). The unpaired Student’s *t*-test (two-tailed) was used for comparing the significance of the expression level differences of the genes between clinical ECC and *Enterobacter cloacae* ATCC 700323. Results with *P*-values < 0.05 were considered to indicate statistical significance.

## Supplementary Information

**Additional file 1: Table S1.** Primer sequence, production size and annealing temperature used in this study

**Additional file 2: Table S2.** MICs of carbapenem and colistin to 19 ECC strains.

**Additional file 3: Table S3.** The results of efflux inhibitors assay

## Data Availability

The datasets used and analysed during the current study are available from the corresponding author on reasonable request. And the sequences of the following type strains used in analysis based on *hsp60* were retrieved from the GenBank database (the information in parentheses is the strain designation, GenBank accession number): *E. asburiae* (ATCC 35953, AJ417141), *E. kobei* (ATCC BAA260, AJ567899), *E. cloacae subsp. dissolvens* (ATCC 23373, AJ417143), *E. ludwigii* (EN-119, AJ417114), *E. hormaechei subsp. oharae* (EN-314, AJ543782), *E. hormaechei subsp. hormaeche*i (ATCC 49162, AJ417108), *E. hormaechei subsp. steigerwaltii* (CIP108489, AJ543908), *E. nimipressuralis* (ATCC 9912, AJ567900), *E. cancerogenus* (ATCC 33241, AJ567895), *E. amnigenus* (ATCC 3072, AJ567894), *E. cowanii* (ATCC 107300 T, AJ567896), *E. gergoviae* (ATCC 33028, AJ567897), *E. pyrinus* (ATCC 49851, AJ567901), *C. sakazaki* (ATCC 29544, AJ567902), *E. aerogenes* (AB008141), and *E. cloacae subsp. cloacae* (ATCC 13049, AJ417142).

## Abbreviations

ECC: *Enterobacter cloacae* complex
PCR: Polymerase chain reaction
ICU: Intensive care unit
MDR: Multi-drug resistance
ESKAPE: *Enterococcus faecalis, Staphylococcus aureus*, *Klebsiella pneumoniae*, *Acinetobacter baumannii*, *Pseudomonas aeruginosa*
ESBL: Extended-spectrum β-lactamase
MIC: Minimum inhibitory concentrations
CCCP: Carbonyl cyanide m-chlorophenylhydrazone
MALDI-TOF MS: Matrix-assisted laser desorption/ionization time of flight mass spectrometry
CLSI: Clinical and Laboratory Standards Institute
EUCAST: European Committee on Antimicrobial Susceptibility Testing
mCIM: Modified carbapenem inactivation methods
qPCR: Quantitative real-time PCR
