# Supplementary material for: Characterization of resistance mechanisms of Enterobacter cloacae Complex co-resistant to carbapenem and colistin
Source: BMC Microbiol. 2021 Jul 8;21:208. doi: 10.1186/s12866-021-02250-x (PMC8268410; doi:10.1186/s12866-021-02250-x)
Supplement: Supplementary file 1 — Additional file 1: Table S1. Primer sequence, production size and annealing temperature used in this study [file 12866_2021_2250_MOESM1_ESM.docx]

| **Table S1 Primer sequence, production size and annealing temperature used in this study** | | | | |
| --- | --- | --- | --- | --- |
| **Primer** | | **suquence** | **Size(bp)** | **Annealing(℃)** |
| *bla*_KPC_ | **F: TCGCCGTCTAGTTCTGCTGTCTT R: CCGCGCAGACTCCTAGCCTAA** | | 965 | 55 |
| *bla*_NDM_ | **F：TCACCGAGATTGCCGAGCGA**  **R：GGGCAGTCGCTTCCAACGGT** | | 457 | 55 |
| *bla*_IMP_ | **F：ATGAGCAAGTTATCTGTATTCTTTAT**  **R：TTAGTTGCTTAGTTTTGATGGTTT** | | 741 | 55 |
| *bla*_VIM_ | **F：GGTCGCATATCGCAACGCAGT**  **R：CGGCGACTGAGCGATTTTTG** | | 636 | 55 |
| *bla*_IMI_ | **F：CCATTCACCCATCACAAC**  **R：CTACCGCATAATCATTTGC** | | 440 | 55 |
| *bla*_SPM_ | **F：CTGCTTGGATTCATGGGCGC**  **R：CCTTTTCCGCGACCTTGATC** | | 783 | 55 |
| *bla*_OXA-23_ | **F：ACTTGCTATGTGGTTGCTTCTCTT**  **R：TTCAGCTGTTTTAATGATTTCATCA** | | 797 | 55 |
| *bla*_OXA-24_ | **F：CGATCAGAATGTTCAAGCGC**  **R：ACGATTCTCCCCTCTGCGC** | | 559 | 55 |
| *bla*_OXA-48_ | **F：TTGGTGGCATCGATTATCGG**  **R：GAGCACTTCTTTTGTGATGGC** | | 744 | 55 |
| *bla*_OXA-58_ | **F:CGATCAGAATGTTCAAGCGC**  **R：ACGATTCTCCCCTCTGCGC** | | 529 | 55 |
| *bla*_Nmc-A_ | **F: TGGTGTCACGCTTTAGACAC**  **R1: ACCATGTCTGATAGGTTTCC** | | 459 | 55 |
| *bla*_FRI-1_ | **F: TGAACTCATTCGCCTCTCAG**  **R: CTGCTTCGTCATGTTTGTCG** | | 734 | 55 |
| *bla*_BIC_ | **F: TATGCAGCTCCTTTAAGGGC**  **R: TCATTGGCGGTGCCGTACAC** | | 537 | 55 |
| *bla*_GIM_ | **F: TCGACACACCTTGGTCTGAA**  **R: AACTTCCAACTTTGCCATGC** | | 477 | 55 |
| *bla*_SME_ | **F:ACTTTGATGGGAGGATTGGC**  **R:ACGAATTCGAGATCACCAG** | | 551 | 55 |
| *bla*_AIM_ | **F: CTGAAGGTGTACGGAAACAC**  **R: GTTCGGCCACCTCGAATTG** | | 322 | 55 |
| *bla*_DIM_ | **F: GCTTGTCTTCGCTTGCTAACG**  **R: CGTTCGGCTGGATTGATTTG** | | 699 | 55 |
| *bla*_SIM_ | **F: TACAAGGGATTCGGCATCG**  **R: TAATGGCCTGTTCCCATGTG** | | 570 | 55 |
| *bla*_GES_ | **F: GGTGCAGCTTAGCGACAATG**  **R: GCGTAATCTCTCTCCTGGGC** | | 467 | 55 |
| *bla*_CTX-M-1_ | **F: AAAAATCACTGCGTCAGTTCAC**  **R: ACAAACCGTTGGTGACGATT** | | 867 | 55 |
| *bla*_CTX-M-9_ | **F: TAT TGGGAGTTTGAGATGGT**  **R: TCCTTCAACTCA GCAAAAGT** | | 933 | 50 |
| *bla*_CTX-M-14_ | **F:** **SCSATGTGCAGYACCAGTAA**  **R:** **ACYTTACTGGTRCTGCACAT** | | 853 | 55 |
| *bla*_SHV_ | **F: AGCCGCTTGAGCAAATTAAAC**  **R: ATCCCGCAGATAAATCACCAC** | | 713 | 60 |
| *bla*_TEM_ | **F: CATTTCCGTGTCGCCCTTATTC**  **R: CGTTCATCCATAGTTGCCTGAC** | | 800 | 60 |
| *ecr* | **F: TATGGTGCATCGAAGGTCGG**  **R: CTACAATGCCTGCCTCGACT** | | 150 | 55 |
| *mcr-1* | **F：GCAGCATACTTCTGTGTGGTAC**  **R：ACAAAGCCGAGATTGTCCGCG** | | 1500 | 55 |
| *mcr-2* | **F:** **CAAGTGTGTTGGTCGCAGTT** | | 715 | 55 |
|  | **R:** **TCTAGCCCGACAAGCATACC** | |  |  |
| *mcr-3* | **F:** **AAATAAAAATTGTTCCGCTTATG**  **R:** **AATGGAGATCCCCGTTTTT** | | 929 | 55 |
| *mcr-4* | **F:** **TCACTTTCATCACTGCGTTG**  **R:** **TTGGTCCATGACTACCAATG** | | 1116 | 55 |
| *mcr-5* | **F:** **ATGCGGTTGTCTGCATTTATC**  **R:** **TCATTGTGGTTGTCCTTTTCTG** | | 1644 | 55 |
| *ompC* | **F:** **GCGACCAGACCTACATGCGT**  **R:** **TTCGTTCTCACCAGAGTTACCCT** | | / | 55 |
| *opmF* | **F:** **TCCCTGCCCTGCTGGTAG**  **R:** **TCCCTGCCCTGCTGGTAG** | | / | 55 |
| *acrA* | **F:** **AACGAACATACCTGGCAACA**  **R:** **GTGGAGCTGGTCACTAACGA** | | / | 55 |
| *acrB* | **F:** **CGATAACCTGATGTACATGTCC**  **R:** **CCGACAACCATCAGGAAGCT** | | / | 55 |
| *rpoB* | **F: AAGGCGAATCCAGCTTGTTCAGC**  **R:** **TGACGTTGCATGTTCGCACCCATCA** | | / | 55 |
