# Supplementary material for: Characterization of resistance mechanisms of Enterobacter cloacae Complex co-resistant to carbapenem and colistin
Source: BMC Microbiol. 2021 Jul 8;21:208. doi: 10.1186/s12866-021-02250-x (PMC8268410; doi:10.1186/s12866-021-02250-x)
Supplement: Supplementary file 2 — Additional file 2: Table S2. MICs of carbapenem and colistin to 19 ECC strains. [file 12866_2021_2250_MOESM2_ESM.docx]

**Table S2 MICs of carbapenem and colistin to 19 ECC strains.**

| Time（year） | Strian | MIC（μg/mL） | | | |
| --- | --- | --- | --- | --- | --- |
|  |  | MEM | IMP | ETP | COL |
| 2011 | Y541 | ≤0.125 (S) | 0.5 (S) | 2 (R) | 8 (R) |
| 2013 | CG175 | 64 (R) | 64 (R) | ﹥128 (R) | ﹥64 (R) |
| 2015 | CG648 | 4 (R) | 4 (R) | 4 (R) | 4 (R) |
| 2015 | CG737 | ≤0.125 (S) | 0.5 (S) | 4 (R) | ﹥64 (R) |
| 2015 | CG741 | ≤0.125 (S) | 0.25 (S) | 4 (R) | 64 (R) |
| 2015 | CG701 | 0.25 (S) | 2 (I) | 2 (R) | ﹥64 (R) |
| 2016 | CG864 | ≤0.125 (S) | 0.5 (S) | 8 (R) | ﹥64 (R) |
| 2016 | CG884 | ≤0.125 (S) | 0.5 (S) | 2 (R) | 32 (R) |
| 2016 | CG934 | ≤0.125 (S) | 0.25 (S) | 2 (R) | ﹥64 (R) |
| 2016 | CG1038 | 1 (S) | 4 (R) | 2 (R) | 4 (R) |
| 2016 | CG1048 | ≤0.125 (S) | 0.5 (S) | 4 (R) | 32 (R) |
| 2016 | CG1050 | ≤0.125 (S) | 0.5 (S) | 2 (R) | 16 (R) |
| 2016 | CG1051 | ≤0.125 (S) | 0.25 (S) | 4 (R) | ﹥64 (R) |
| 2017 | CG1249 | 4 (R) | 0.5 (S) | 16 (R) | ﹥64 (R) |
| 2017 | CG1400 | 2 (I) | 1 (S) | 2 (R) | ﹥64 (R) |
| 2018 | CG1479 | 8 (R) | 16 (R) | 128 (R) | ﹥64 (R) |
| 2018 | CG1506 | 2 (I) | 8 (R) | 4 (R) | ﹥64 (R) |
| 2018 | CG1574 | 1 (S) | 2 (I) | 8 (R) | ﹥64 (R) |
| 2018 | CG1819 | 8 (R) | 4 (R) | 32 (R) | ﹥64 (R) |

MEM, meropenem; IMP, imipenem; ETP, ertapenem; COL, colistin; S, sensitive; R, resistant。
