# Supplementary material for: Characterization of resistance mechanisms of Enterobacter cloacae Complex co-resistant to carbapenem and colistin
Source: BMC Microbiol. 2021 Jul 8;21:208. doi: 10.1186/s12866-021-02250-x (PMC8268410; doi:10.1186/s12866-021-02250-x)
Supplement: Supplementary file 3 — Additional file 3: Table S3. The results of efflux inhibitors assay [file 12866_2021_2250_MOESM3_ESM.docx]

**Table S3** **The results of efflux inhibitors assay**

| Strains | MIC (μg/L) | | | | | | | |
| --- | --- | --- | --- | --- | --- | --- | --- | --- |
|  | ETP | ETP+CCCP | ETP+omeprazole | ETP+reserpine | COL | COL+CCCP | COL+omeprazole | COL+reserpine |
| CG701 | 2 | 1 | 1 | 2 | >64 | 0.125 | 0.5 | 64 |
| CG1050 | 2 | 2 | 2 | 1 | 16 | 0.25 | 0.5 | 16 |
| CG1051 | 4 | 4 | 4 | 2 | >64 | 0.25 | 0.5 | >64 |
| CG648 | 4 | 2 | 4 | 2 | 4 | 0.06 | 1 | 4 |
| CG1479 | 128 | 64 | 128 | 128 | >64 | 0.125 | 0.5 | 64 |
| CG1574 | 8 | 4 | 8 | 8 | >64 | 0.125 | 0.25 | >64 |
| Y541 | 2 | 2 | 2 | 2 | 8 | 0.125 | 8 | 8 |
| CG737 | 4 | 2 | 4 | 4 | >64 | 0.125 | 0.25 | >64 |
| CG741 | 4 | 2 | 4 | 2 | 64 | 0.125 | 0.25 | 64 |
| CG884 | 2 | 2 | 2 | 2 | 32 | 0.125 | 1 | 32 |
| CG1038 | 2 | 2 | 2 | 1 | 4 | 0.06 | 0.125 | 4 |
| CG1506 | 4 | 2 | 4 | 4 | >64 | 0.25 | 0.5 | >64 |
| CG864 | 8 | 8 | 4 | 4 | >64 | 0.125 | 16 | >64 |
| CG934 | 2 | 2 | 2 | 1 | >64 | 0.125 | 0.5 | >64 |
| CG1400 | 2 | 2 | 2 | 2 | >64 | 0.125 | 0.25 | >64 |
| CG1048 | 4 | 4 | 4 | 2 | 32 | 0.5 | 2 | 32 |
| CG1249 | 16 | 16 | 16 | 16 | >64 | 1 | >64 | >64 |
| CG175 | >128 | >128 | >128 | >128 | >64 | 0.06 | 1 | 0.5 |
| CG1819 | 32 | 32 | 32 | 32 | >64 | 0.125 | 0.5 | >64 |

ETP, ertapenem; COL, colistin.
